# Comparative Analysis of Mixed Reality and PowerPoint in Education: Tailoring Learning Approaches to Cognitive Profiles

**DOI:** 10.3390/s24165138

**Published:** 2024-08-08

**Authors:** Radu Emanuil Petruse, Valentin Grecu, Marius-Bogdan Chiliban, Elena-Teodora Tâlvan

**Affiliations:** 1 Faculty of Engineering, Lucian Blaga University of Sibiu, 550024 Sibiu, Romania; radu.petruse@ulbsibiu.ro (R.E.P.); bogdan.chiliban@ulbsibiu.ro (M.-B.C.); 2 Faculty of Medicine, Lucian Blaga University of Sibiu, 550024 Sibiu, Romania; elena.talvan@ulbsibiu.ro

**Keywords:** cognitive ability, education, mixed reality, PowerPoint, teaching methods

## Abstract

The term immersive technology refers to various types of technologies and perspectives that are constantly changing and developing. It can be used for different purposes and domains such as education, healthcare, entertainment, arts, and engineering. This paper aims to compare the effectiveness of immersive technologies used in education, namely mixed reality, generated with Microsoft HoloLens 2, with traditional teaching methods. The experiment involves comparing two groups of students who received different training methods: the first group saw a PowerPoint slide with an image of the human muscular system, while the second group saw a 3D hologram of the human body that showed the same muscle groups as in the PowerPoint (PPT). By integrating the Intelligence Quotient (IQ) levels of the participants as a predictive variable, the study sought to ascertain whether the incorporation of mixed reality technology could significantly influence the learning outcomes and retention capabilities of the learners. This investigation was designed to contribute to the evolving pedagogical landscape by providing empirical evidence on the potential benefits of advanced educational technologies in diverse learning environments. The main finding of this study indicates that while MR has potential, its effectiveness is closely tied to its interactivity. In cases where the content remains static and non-interactive, MR does not significantly enhance in-formation retention compared to traditional PPT methods. Additionally, the study highlights that instructional strategies should be adapted to individual cognitive profiles, as the technology type (MR or PPT) alone does not significantly impact learning outcomes when the information presented is identical.

## 1. Introduction

Educational technology or EdTech is a systematic approach to educational processes and resources to improve student performance. This allows for identifying students’ needs and adapting the instructive-educational process to them to ensure student development. Educational technology is a field that combines educational hardware, software, theory, and practice to enhance the quality and effectiveness of education [1]. It is also an industry that creates and provides various technological tools and environments for education [2]. One of the emerging forms of educational technology is immersive technology, which includes virtual reality (VR), augmented reality (AR), and mixed reality (MR) [3]. 

Immersive technology offers lifelike experiences [4], enhancing learning in healthcare [5], education [6], and crisis response [7,8]. These tools engage users’ thinking, feelings, and actions, leading to better learning outcomes.

While immersive technology is diverse and ever-changing, with applications across education, healthcare, and more [4,9,10,11,12], it is set to grow, bringing new opportunities and hurdles. It is effective for learning, offering tailored feedback, and engaging experiences. Yet, it faces technical, ethical, and access challenges that need addressing through further research [13,14,15].

To differentiate these three technologies, we need to look at them from the user’s perspective (see Table 1 and Table 2). In VR, the user is totally immersed in a complete simulation environment in which they have the possibility to interact only with the virtual environment, without having the possibility to relate to the elements in the physical environment. To use VR, the minimum technology required consists of head-mounted virtual reality glasses and a computer that will carry out the graphics processing. Instead, AR adds digital elements to the real world via a device such as a smartphone or tablet through which the user observes the real environment. MR is a technology that combines elements of virtual reality and augmented reality by using “glasses” that have a transparent screen that allows viewing 3D digital objects, like holograms, and interaction with these digital objects is mostly performed through hand gestures, voice commands, or simply fixing the gaze at a specific point.

Looking at immersive technology on the Gartner Hype Cycle [18], we can see that it is located at the border between existing technologies that we can apply in higher education and technologies that are already in use. 

Mixed reality (MR) in education offers significant advantages over traditional teaching methods, as evidenced by various studies. Ali et al. found that medical students showed high satisfaction levels with MR models for anatomy learning [19], while Minty et al. demonstrated that HoloLens 2 is a valid and robust tool for objectively assessing clinical competency in medical students through Objective Structured Clinical Examinations (OSCEs) [20]. Pregowska et al. highlighted how MR, especially through devices like HoloLens 2, can revolutionize education by providing immersive experiences in subjects like medicine, anatomy, and biochemistry, enhancing both remote and traditional learning modalities [21]. Additionally, Daling et al. emphasized the usefulness of MR in remote teaching, showing that it improves students’ understanding and provides a realistic experience compared to traditional methods, particularly in practical fields like mining engineering [22]. Overall, MR in education shows great promise in enhancing learning outcomes and student engagement when compared to traditional approaches. Furthermore, the use of mixed reality gadgets in educational settings has shown promising results, with applications designed for training and education purposes demonstrating benefits over traditional teaching methods, as observed in experiments with secondary school students [23]. 

While existing research emphasizes the potential of mixed reality (MR) to enhance teaching methods and improve information retention, no prior study has specifically explored the interaction between IQ and instructional approaches. Our approach aims to investigate the individual impact of participants’ IQ levels on learning outcomes. Given the absence of similar studies in the literature, this paper examines the implications of using mixed reality technologies in the learning process, including their effects on students’ memory. This study evaluates the effectiveness of immersive technologies, with a particular focus on mixed reality, in educational contexts compared to traditional teaching methods, while also considering participants’ IQ as a distinguishing factor

In traditional education systems, PowerPoint presentations are commonly utilized due to their accessible interface and customizable templates. However, this method has its limitations, including a slow adaptation to new technologies and a potential restriction on active learning due to an over-reliance on slides. Nevertheless, the incorporation of mixed reality in education is still in its early stages, requiring substantial investment in equipment and training. Furthermore, it is important to note that not all educational content may be appropriate or effective when presented in a mixed reality format.

The relation between MR and IQ in the educational environment presents an exciting opportunity for research. One of the research questions of this study is “How do MR and IQ interplay to optimize educational efficacy?” This analysis is fundamental as it explores the hypothesis that MR, when aligned with the learner’s IQ, can significantly enhance the effectiveness of educational outcomes.

MR technology, with its immersive and interactive capabilities, stands as an educational innovation [24,25], offering a stark contrast to the passive learning modes associated with traditional tools like PowerPoint presentations [26,27]. The integration of MR in education promises a more dynamic and engaging learning environment, where students can interact with complex concepts in a three-dimensional space, fostering deeper understanding and retention [9,11].

However, the effectiveness of MR is not uniform across all learners; it is hypothesized that individual differences in cognitive abilities, as indicated by IQ, may influence the degree to which students benefit from MR-based education [28,29,30]. This study posits that a student’s IQ could be a critical factor in determining the optimal level of MR immersion and interactivity, thus maximizing educational efficacy.

By investigating the relation between MR and IQ, this research aims to provide empirical evidence on the customization of educational technologies to individual cognitive profiles. This could lead to a paradigm shift in educational practices, moving away from a one-size-fits-all approach to a more nuanced, intelligence-informed pedagogy. The findings could have far-reaching implications for the design of future educational tools and curricula, ensuring that the transformative potential of MR is fully realized in enhancing learning experiences.

## 2. Immersive Technologies in Education

The term immersive technology refers to the integration of virtual content with the physical environment in a way that allows the user to naturally engage with mixed reality. The origin of immersive technology can be traced back nearly 60 years, when the first immersive prototype of human–computer interaction, the “Human Machine Graphic Communication System”, was built by Sutherland [31]. Since then, different types of immersive technologies have emerged, such as VR, AR, MR, holography, telepresence, digital twins, and FPV drone flight [4].

However, there is no consensus on the definition of immersive technology among researchers. Some researchers focus on the quality and quantity of sensory information provided by technology, such as Slater [32], which sees immersive technology as a technology that provides users with a high quality or volume of sensory information. Other researchers emphasize the immersiveness of the technology, such as H.-G. Lee, Chung, and Lee [33], who perceive immersive technology as technology that makes the line between the real world and the virtual world blur, creating a sense of immersion. A third group of researchers considers both aspects of sensory information and immersion, such as Díaz-López et al. [34], which define immersive technology as creating a realistic digital landscape that allows users to feel as if they are indoors and interacting with that environment.

These technologies create simulated or enhanced environments that allow learners to interact with realistic scenarios and objects [35,36]. Immersive technologies can support effective teaching methods that align with learning goals and outcomes [4,11,37,38]. They may also encourage constructivist and experiential learning approaches that allow students to build their own knowledge, learn by doing, develop creativity, and understand abstract concepts [39]. As stated by the Association for Medical Education in Europe, “Projects for effective medical e-learning must reflect the dynamics and details of real-world practice, as well as provide effective learning opportunities” [40,41]. Therefore, immersive technologies can provide valuable opportunities for medical education and training, as well as other disciplines that require practical approaches and problem-solving skills [3,4,42,43].

The use of such immersive technologies has intensified in education, particularly in health and science [3,4,10,44]. These technologies offer students the opportunity to explore complex topics and scenarios in a realistic and interactive way, which can improve their learning outcomes compared to traditional methods such as lectures, textbooks, or slideshows. However, evidence on the effectiveness of immersive technologies in education is still limited and inconsistent [9,38,45,46]. 

**Figure 1:**
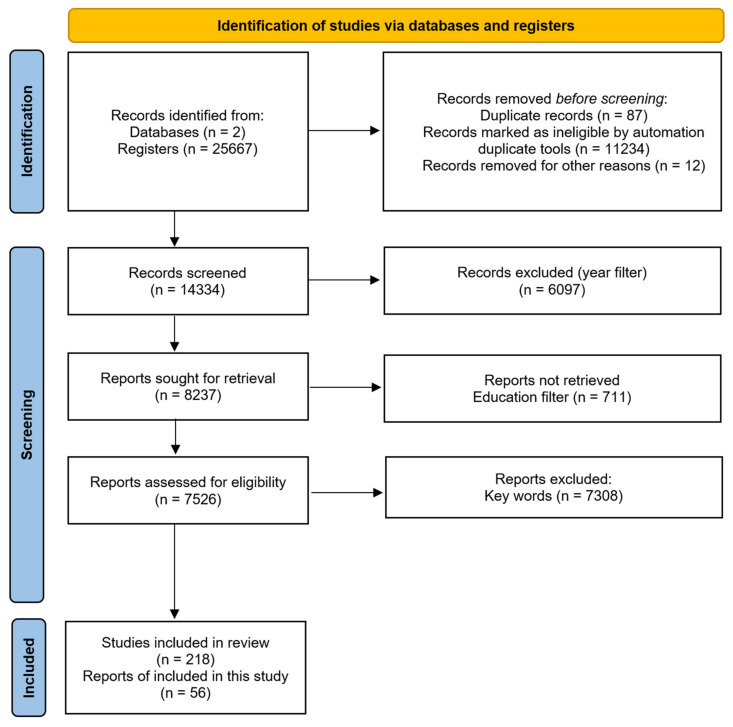
PRISMA flow diagram [47].

In order to establish a comprehensive research overview, the development of the Preferred Reporting Items for Systematic Reviews and Meta-Analysis (PRISMA) Flow Diagram was essential. This diagram, depicted in Figure 1, serves as a visual representation of the process undertaken during the research analysis.

To investigate the advancements and evolution of immersive technologies implemented in educational settings, a thorough exploration was conducted utilizing the Scopus and Web of Science databases. The search strategy involved employing specific keywords such as mixed reality, augmented reality, virtual reality, and education. Initially, a broad search across all article fields yielded a total of 25,667 outcomes, which were then subjected to the PRISMA methodology, resulting in the identification of 218 relevant articles for further examination.

Subsequently, the pool of relevant articles was utilized to construct a network map using the VosViewer 1.16.20 software tool. This network map offers an overview of the primary subjects and emerging trends within the topic of immersive technologies in education.

The visual representations within the network map are derived from the most frequently utilized keywords within the literature. A specific threshold of a minimum of five occurrences per keyword was established for inclusion in the visualization. These threshold criteria were established based on data extracted from the Web of Science (WoS) and Scopus databases. By implementing such a threshold, the visualization process focused on the most prevalent and potentially significant terms in the domain of immersive technologies in education. This approach effectively filters out less common terms, thereby enhancing the clarity and interpretability of the visualization.

**Figure 2:**
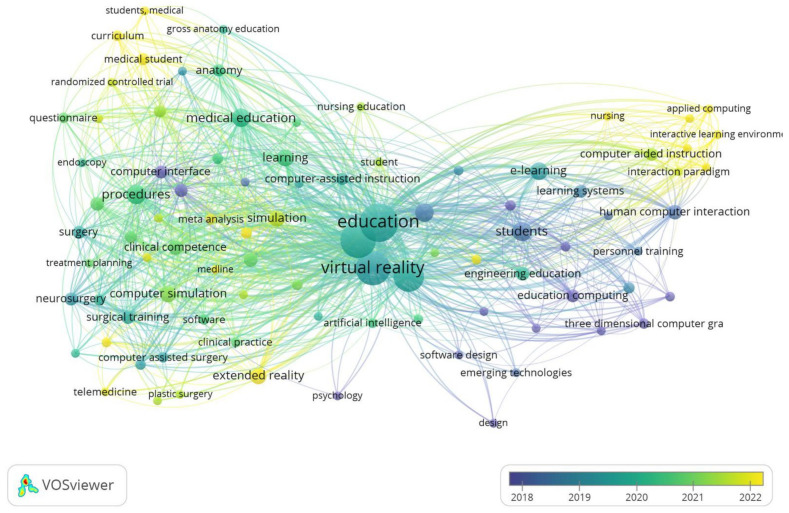
VOSViewer network visualization map.

Illustrated in Figure 2, the VOSViewer Network Visualization Map showcases a snapshot of the prevailing trends and terminologies relevant to immersive technologies in education. Key terms such as “augmented reality”, “mixed reality”, “virtual reality”, and “education” are displayed in the center of the map, underscoring their importance and frequency within the field, thus validating the initial database search. Moreover, trends towards interactive learning environments are observable through keywords highlighted in light green and yellow, emphasizing newer research directions. Additionally, we can observe that immersive technologies have been mostly applied for medical education, as suggested by keywords such as “medical education”, “students”, and “anatomy”.

These visual depictions serve to offer an overview of the primary subjects and their interconnectedness within this field.

The current state of the art in this area is based on a growing body of research investigating the effects of AR, VR, and MR on various aspects of learning, such as motivation, commitment, self-efficacy, cognitive task, self-regulation, and knowledge acquisition and transfer [4,9,10,44]. 

A systematic review by Hamilton et al. [9] examined 29 experimental studies published since 2013 in which quantitative learning outcomes using immersive VR based on head mounted display were compared to less immersive pedagogical methods. The study found that most studies have demonstrated benefits in terms of learning outcomes when using immersive VR compared to less immersive learning methods. A smaller number of studies did not find any significant advantage, regardless of the pedagogical method used. Only two studies found clear harmful effects of immersive VR use [48,49]. However, this analysis also highlighted some limitations of existing research, such as short intervention times, lack of information retention measures, focus on scientific topics, and inadequate evaluation methods [50,51].

Another systematic review conducted by Ryan et al. [3] evaluated 29 randomized controlled trials (N = 2722 students) comparing traditional learning methods with VR, AR, or MR for the education of medical and nursing students. The analysis found that knowledge acquisition was equal when immersive technologies were compared to traditional ways of learning. However, the learning experience has grown with immersive technologies. This study also reported that learning outcomes such as student satisfaction, self-efficacy, and engagement all increased with the use of immersive technology, suggesting that it is an optimal tool for education.

A third systematic review by Butt et al. [37] looked at 18 studies (N = 1090 participants) that investigated the use of immersive VR for training healthcare professionals in various clinical skills. The analysis found that immersive VR training was associated with improved performance, knowledge retention, and confidence compared to traditional training methods. This study identified some challenges and barriers to implementing immersive VR training in healthcare education, such as technical issues, cost, affordability, and ethical concerns.

These studies indicate that immersive technologies have the potential to improve students’ learning outcomes in health and science education by providing them with a rich, interactive, engaging, and safe learning experience while emphasizing the transferability of skills in clinical settings. However, more rigorous and consistent research is needed to establish the optimal design, implementation, and evaluation of immersive technology-based interventions in education.

However, there are still many gaps and challenges in immersive learning research, such as the lack of rigorous experimental studies, diversity of definitions and measures, ethical and practical aspects of implementing immersive technologies in real-world contexts, and the need for closer interdisciplinary collaboration between researchers and practitioners [24,52,53]. Furthermore, there is a lack of research on how individual differences between learners, such as their IQ, may influence their immersive learning experience [54,55,56]. IQ is a measure of general cognitive ability that can affect various aspects of learning, such as memory, problem solving, reasoning, and metacognition [57]. It is possible that IQ interacts with immersion level, controls, and representative fidelity of immersive technologies to affect learners’ presence and cognitive load [58].

## 3. IQ as a Predictor of Academic Success: Evaluating the Evidence

The relationship between IQ and academic performance is a subject of considerable interest in educational psychology. Research indicates that IQ scores are a strong predictor of academic success, but they are not the sole factor [29,59,60]. Cognitive ability, as measured by IQ tests, often correlates with better academic outcomes because it reflects a person’s ability to think abstractly, solve problems, and grasp complex ideas [61,62].

However, studies also suggest that non-cognitive factors such as personality traits, motivation, and learning strategies can significantly influence academic performance [59,63]. For instance, conscientiousness has been linked to better study habits and academic achievement, while traits like curiosity and openness may foster a love for learning that transcends raw cognitive ability [64].

Moreover, the environment plays a crucial role in shaping academic outcomes [65,66]. A supportive educational setting, access to resources, and quality instruction can enhance the academic performance of students, regardless of their IQ. Socioeconomic status and parental involvement are also critical factors that can impact educational achievement.

The relationship between IQ and academic performance has been extensively studied, revealing a multifaceted connection. Rohde and Thompson [61] found that general cognitive ability, measured by tools like the Raven’s Advanced Progressive Matrices and the Mill Hill Vocabulary Scales, was a significant predictor of academic achievement, even when controlling for specific cognitive abilities. Rushton et al. [28] demonstrated that performance on the Raven’s Matrices correlated with academic success among diverse student groups, suggesting that general intelligence plays a role across different cultures.

Ablard and Mills [67] highlighted the Raven’s Matrices as effective for identifying academically talented students, indicating that higher-order cognitive abilities are linked to academic competency. Zax and Rees [63] explored the impact of IQ on earnings and found that while IQ does affect earnings, its influence is less significant when accounting for family and academic performance. Heaven and Ciarrochi [68] argued that intellect, a component of cognitive ability, is associated with higher academic performance, particularly among those with high ability.

Mayes et al. [29] identified IQ as the best single predictor of academic achievement, with other neuropsychological tests contributing to the prediction of specific academic skills. Chamorro-Premuzic and Furnham [59] found that personality traits and learning approaches also play a role in academic performance, with ability effects mediated by these factors. Byington and Felps [69] provided a sociological perspective, suggesting that the predictive power of IQ on job performance may be due to institutional factors that grant individuals with high IQ scores greater access to developmental resources.

Iqbal et al. [30] examined medical students and found a positive relationship between IQ and academic performance, reinforcing the notion that cognitive ability is a crucial factor in educational success. Collectively, these studies underscore the importance of IQ in academic achievement while also recognizing the influence of personality, learning approaches, and sociocultural factors.

Lastly, Allor et al. [70] established that tailoring pedagogical strategies and the broader educational framework to align with students’ intellectual capacities, thereby catering to their specific requirements, can significantly enhance the efficacy of learning outcomes. In summary, while IQ is an important aspect of academic performance, it is part of a broader constellation of factors that include individual characteristics, environmental influences, and the interplay between them. This holistic view acknowledges that intelligence is not fixed and that a variety of elements contribute to academic success [60].

## 4. Experimental Methodology

This paper aims to compare the effectiveness of immersive technologies used in education, namely mixed reality, generated with Microsoft HoloLens 2, with traditional teaching methods. This study was conducted on N = 98 students of Lucian Blaga University of Sibiu, from the Faculty of Engineering and the Faculty of Social and Human Sciences. The selected students were surveyed beforehand to identify if they had any knowledge of the didactic material, which affected the result obtained. 

### 4.1. Research Hypothesis

After reviewing the existing literature, we have developed a research hypothesis for our study, which speculates the following: “Educational experiences enhanced by immersive technologies surpass the efficacy of conventional teaching approaches. It is anticipated that students’ learning outcomes will vary depending on the type of instructions received (MR or PPT) and the results will also be influenced by the participant’s IQ score”. Moreover, through this approach, we can also study which instructional method is more suitable to different IQ levels. We plan to validate this hypothesis by examining how different instructional methods and student IQ levels influence learning outcomes within an educational framework that utilizes both traditional and immersive technological approaches.

### 4.2. Justification for Selecting Human Anatomy as the Study Focus

Human anatomy is a foundational subject that supports many fields of study within both the sciences and the humanities. Its complex nature, characterized by complex structures and interdependent systems, poses significant challenges for traditional educational methods. The spatial and three-dimensional aspects of human anatomy often require more than simple images or textual descriptions to be understood effectively. This complexity makes it an ideal candidate for the application of mixed reality (MR) technologies.

MR, with its ability to overlay digital information onto the real world, offers a unique opportunity to augment the learning experience by providing students with interactive, three-dimensional visualizations of anatomical structures. This can lead to a deeper understanding and retention of the subject matter, which is less achievable through conventional two-dimensional teaching aids.

Furthermore, the study of human anatomy serves as a critical litmus test for the effectiveness of MR in education due to its universal relevance and the necessity for precision in learning. Unlike subjects such as mathematics, history, or art, which can be effectively taught using traditional methods like lectures and textbooks, human anatomy requires a more immersive approach to grasp the full scope of the body’s complexity.

By focusing on human anatomy, this study aims to explore the potential of MR to transform educational practices in subjects where traditional methods fall short. The exclusion of other subjects is intentional, as they may not provide the same level of challenge or necessity for three-dimensional comprehension that human anatomy does. This focus allows for a clear assessment of MR’s impact on learning outcomes, particularly when correlated with students’ IQ levels, thereby providing valuable insights into the future of educational technology.

### 4.3. Experiment Execution

Only students without knowledge of body anatomy were included in this study to test the hypothesis of this work, which assumes that the teaching material provided through MR helps in the learning process. This study consists of comparing the learning outcomes of two groups of students who were self-trained using mixed reality and traditional teaching materials.

**Figure 3:**
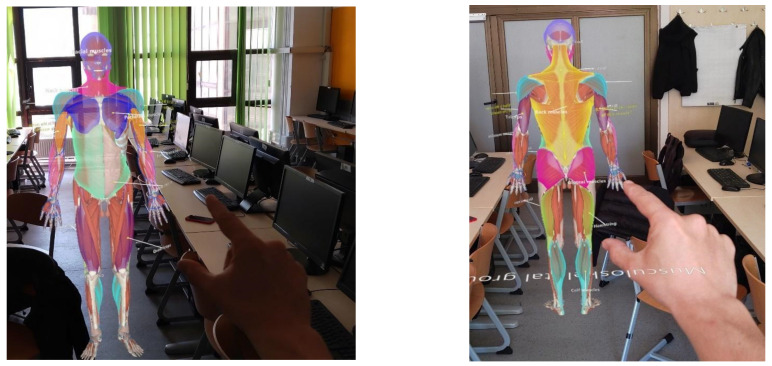
Group 1 teaching material—holographic image of the human muscular system displayed using a HoloLens.

To test whether mixed reality instruction is more effective than PowerPoint instruction for improving student learning outcomes, participants were trained with the two methods mentioned above and then their knowledge was tested using a multiple-choice test. A group of participants received a HoloLens device and were allowed to study the hologram individually for 10 min. The image displayed showed the human muscular system, highlighting muscle groups in different colors, and the names of the most important muscles were shown next to the hologram, as shown in Figure 3.

**Figure 4:**
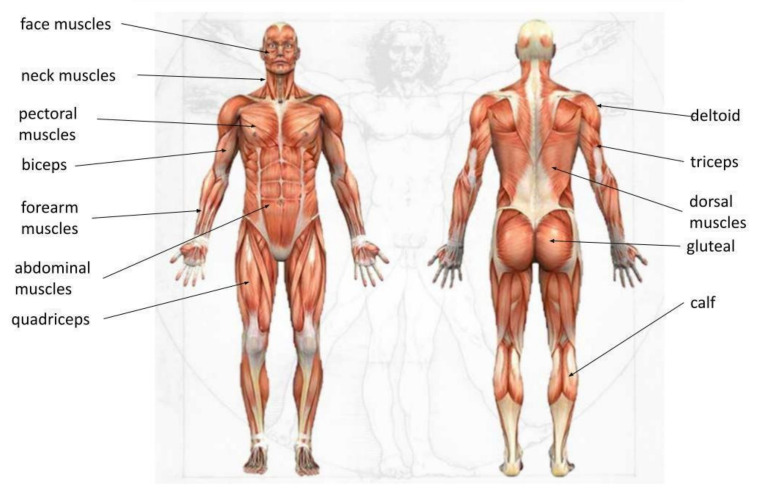
Group 2 teaching material—PowerPoint slide of the human muscular system (source: https://depositphotos.com/stock-photos/muscle.html accessed on 17 August 2023).

The second group of participants was trained with a traditional method. They were presented a PowerPoint slide showing the muscular system, and the same muscle groups were identified according to Figure 4.

In both scenarios, the participants were self-trained. There was no interaction between students and an instructor. The researchers informed the participants that they had 10 min to try to absorb as much information as possible from what they were going to see and that they would be tested afterwards to show how much knowledge they had gathered.

**Figure 5:**
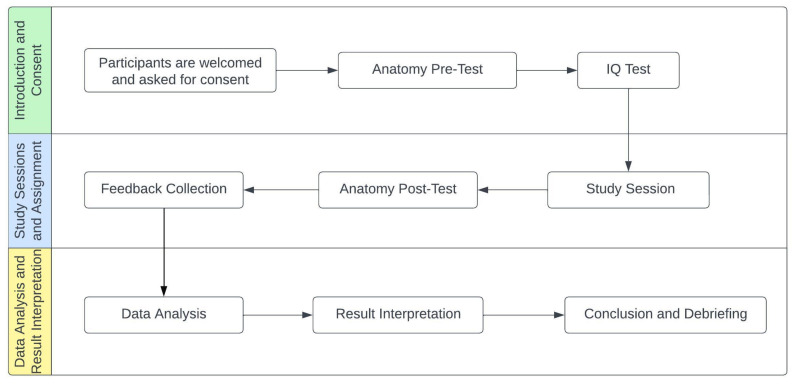
Workflow of the experiment execution.

The experiment was executed as depicted in Figure 5, involving the steps outlined below:

- Introduction and Consent: Participants were welcomed, briefed about the study’s purpose and procedure, and asked to sign a consent form. They also completed a demographic questionnaire, followed by 60 questions aimed at testing participants’ IQ using Raven’s standard progressive matrices.
- Pre-Test: A pre-test was administered to gauge participants’ existing knowledge of the human muscular system. This test was interview-based, with questions about basic muscle anatomy, physiology, and their relative positioning.
- Group Assignment and Study Session: The participants were randomly allocated to the mixed reality (MR) group or the PowerPoint (PPT) group. They were then directed to separate rooms and given 10 min to self-study the material using either the HoloLens 2 device (MR group) or a laptop and projector displaying PowerPoint slides (PPT group).
- Post-Test: Following the study session, the participants took a post-test to assess their learning outcomes. This test consisted of 20 multiple-choice questions covering similar topics to the pre-test. It was administered online via Google Forms and had a 15 min time limit.
- Feedback Collection: After the post-test, the participants were asked to share their learning experience and satisfaction with the material and device used. This feedback was collected through a structured questionnaire.
- Data Analysis and Result Interpretation: The data from the post-test and feedback questionnaire were analyzed. The learning outcomes were measured, and the effectiveness of each teaching method was compared. The influence of participants’ IQ on the effectiveness of the teaching methods was also examined.
- Conclusion and Debriefing: Finally, conclusions were drawn based on the results. The potential implications of the findings for the fields of education and immersive technology were discussed.

### 4.4. Data Analysis

The data collection tool was an online questionnaire, which included 85 questions. There were 5 classification questions, followed by 60 questions aimed at testing the participants’ IQ using Raven’s standard progressive matrices [71], and 20 questions to test their acquired information about the human muscular system. The data were interpreted using Microsoft Excel v2407 and MiniTab 20.

To analyze the data, the authors employed various statistical methods that were appropriate for the research questions and the type of data. First, they computed the Cronbach’s alpha coefficient to evaluate the internal consistency of the items in the anatomy test. A high value of Cronbach’s alpha indicates that the items measure the same construct and are reliable. Second, they performed the Anderson–Darling normality test to check whether the data followed a normal distribution. This is an important assumption for parametric tests, such as *t*-test and regression. Third, they applied the 2-Sample *t* test to compare the means of two independent groups (i.e., the anatomy test scores for the two different instruction methods) and to test whether they were significantly different or not. This is a common statistical technique for testing hypotheses about group differences. Fourth, they conducted a simple linear regression to model the relationship between two continuous variables and to estimate the effect of one variable on the other. For example, they used simple linear regression to examine how IQ score predicted anatomy test score. This is a useful method for exploring causal relationships and predicting outcomes. Fifth, they used multiple regression to analyze the relationship between a dependent variable (i.e., the anatomy test scores) and two or more independent variables (e.g., IQ score and instruction method). This is an extension of simple linear regression that allows for controlling confounding variables and testing interactions. The authors found that both IQ score and instruction method were significant predictors of anatomy test score, and there was a significant interaction between them. This means that the effect of the instruction method on anatomy test score depended on IQ score. The authors chose these statistical methods because they were suitable for answering their research questions and testing their hypotheses. They also followed the guidelines and recommendations from previous studies that used similar methods in educational research [72,73].

### 4.5. Research Questions

In this study, a learning method using MR technology was compared to traditional teaching materials. Students were asked to study the teaching material, and a quasi-experimental study was conducted to test the impact of the learning method on the academic performance of university students and to explore whether their IQ is relevant to the effectiveness of the learning methods tested. This study focused on the following research questions:

1. Do students who use the MR-based learning method exceed the performance of those who learn by the conventional method?
2. Do students with higher IQ perform better using MR-based learning methods compared to using conventional learning methods?

### 4.6. Purpose

This research paper aims to compare the effectiveness of two different training methods: PowerPoint presentations and mixed reality using a HoloLens device. The authors hypothesized that mixed reality devices could enhance students’ cognitive functions by providing them with immersive and interactive learning experiences. To test this hypothesis, they conducted an experimental study with two groups of students who received either PowerPoint or mixed reality training on a specific topic. The learning outcomes of the two different training methods were measured using a 20 questions anatomy test. 

### 4.7. Sample

**Figure 6:**
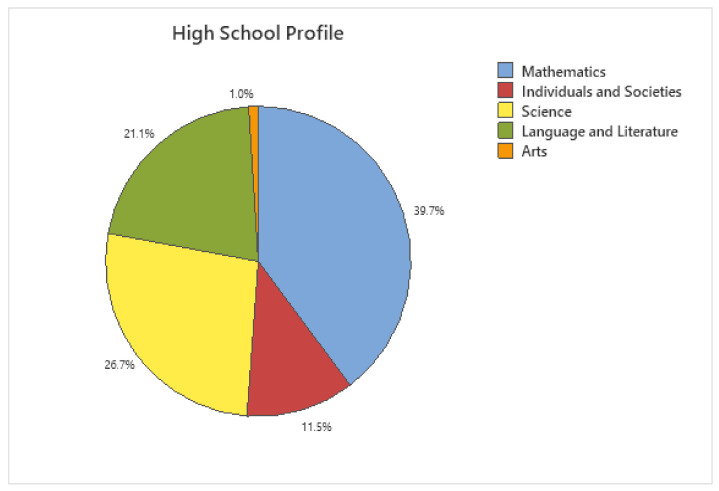
High school profile of participants.

The subjects are students from Lucian Blaga University of Sibiu. They participated in this research voluntarily and were randomly assigned to one of two research groups. There were 98 participants, distributed as shown in Table 3 and Figure 6.

## 5. Results and Interpretation of Data

### 5.1. Validity of the Assessment Method for the Learning Outcomes

In this study, we calculated Cronbach’s alpha for the set of 20 questions that were used to measure the knowledge gained by participants after studying the teaching material (hologram for group 1 and PowerPoint for group 2). Correct answers were scored 1, while wrong answers were marked 0. The results showed that Cronbach’s alpha for the 20 questions was 0.7961, meaning that the questions had a good level of internal consistency and reliability. A detailed calculation of consistency and reliability of the 20 research elements is presented in Table 4.

**Figure 7:**
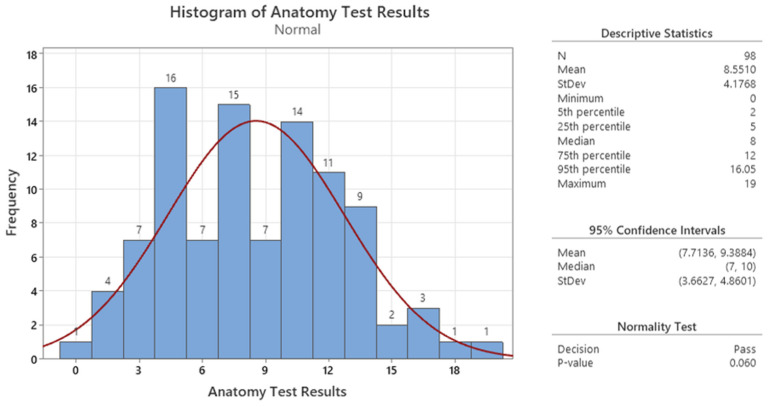
Graphical summary of the anatomy test score.

This implies that the questions were well designed and captured the same basic construction of knowledge about the human muscular system. It also suggests that the participants answered the questions consistently and honestly. Therefore, we can use the scores from the 20 questions as a valid and reliable measure of the knowledge gained in our analysis. Furthermore, upon examining Figure 7, we observe that the data follow a Gaussian distribution. The data are symmetrically distributed, with a nearly constant mean and variation. This provides evidence supporting the accuracy and reliability of the obtained data.

### 5.2. Age Distribution 

**Figure 8:**
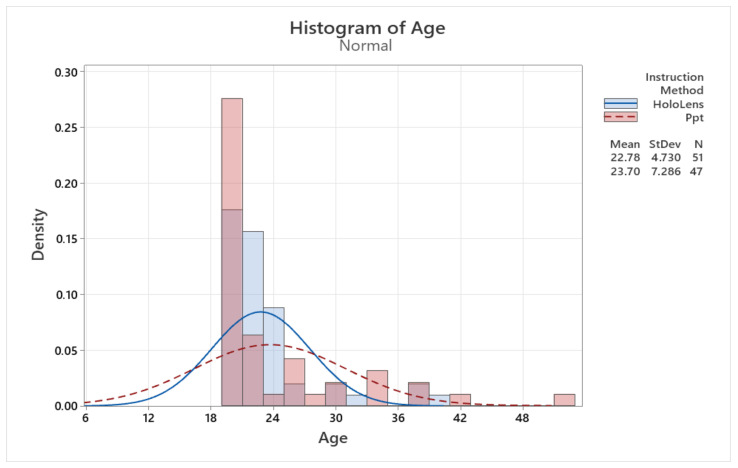
Age distribution of participants.

Figure 8 details the age distribution of the participants, which is non-normal but explained by the environment from which the target group was selected. The age of the participants ranged from 19 to 52 years old, as distance learning students were also involved in the research. The age distribution shown in Figure 8 shows that the two groups had similar profiles. Group 1, which was trained with the HoloLens 2 device, has an average age of 22.78, while Group 2, trained with PowerPoint, has an average age of 23.7.

### 5.3. IQ Score Distribution

**Figure 9:**
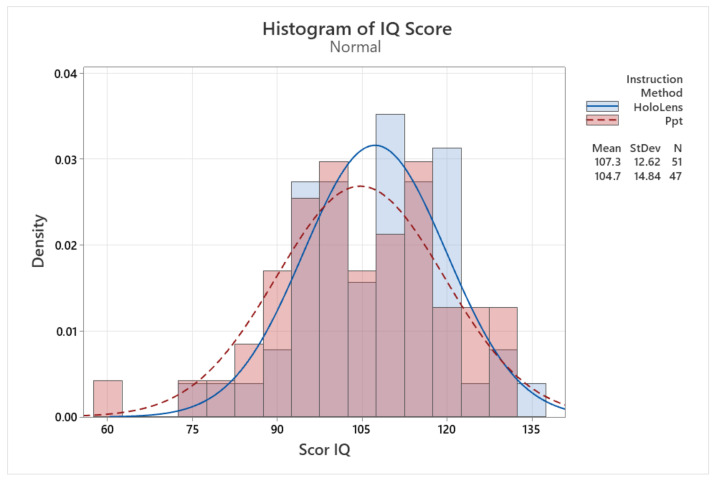
IQ score data distribution.

Each participant was asked to complete an IQ test using Raven’s standard progressive matrices (Figure 9). The test consisted of 60 questions, and the IQ score was calculated according to the authors’ instructions [71]. 

The average IQ score of participants in group 1 is 107.27, with a standard deviation of 12.62 points, while the second research group, trained with PowerPoint, had an average score of 104.74 points, with a standard deviation of 14.84.

The utilization of histograms allows for a graphical depiction of the distribution of IQ scores within each group, aiding in the assessment of potential variations. The overlap observed in Figure 9 indicates that the distributions of IQ scores for both groups exhibit similar patterns, suggesting a lack of substantial disparities in terms of IQ levels.

### 5.4. Anatomy Test Results

**Figure 10:**
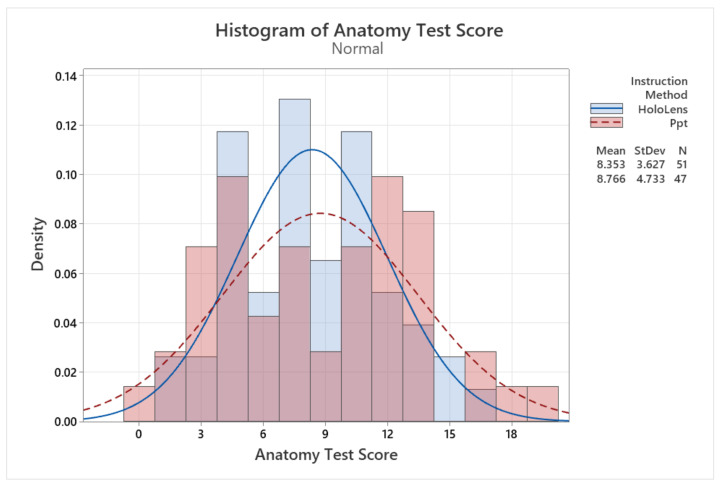
Distribution of data for anatomy test results.

Figure 10 presents the results of the anatomy test, comparing the performance of the two study groups. The histograms for both groups show a normal distribution, with a higher standard deviation for the second group trained with PowerPoint (4.73) compared to the first group trained with HoloLens (3.62). The results indicate that the second group performed slightly better, with an average of 8.76 correct answers out of a maximum of 20, while the first group had an average of 8.35 correct answers.

**Figure 11:**
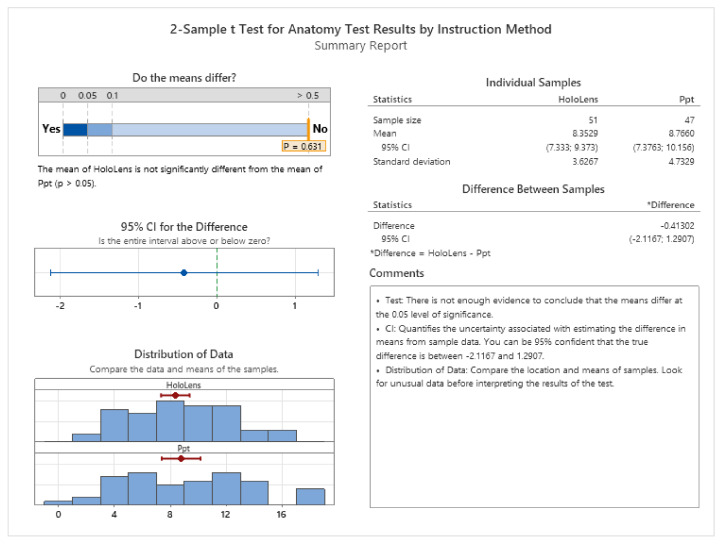
Two-Sample *t* test for the anatomy test results.

The 2-Sample *t* test (see Figure 11) was used to determine whether the means of the anatomy test scores for the two study groups are different.

The analysis shows that the mean of the anatomy test results of students who were instructed with the MR method is not significantly different from the mean of students who were instructed with traditional methods

### 5.5. Relationship between IQ Score, Gender, and Learning Outcomes

**Figure 12:**
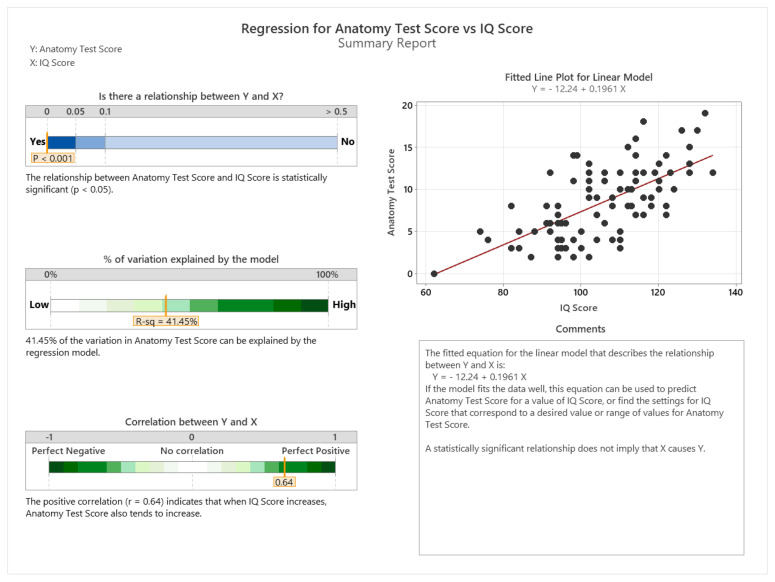
Regression for anatomy test score vs. IQ score.

The regression analysis from Figure 12 provides a comprehensive view of the relationship between students’ IQ scores and their anatomy test results. With a sample size of n = 98, this study has enough data points to yield a precise estimate of this relationship. The narrow confidence interval and small margin of error enhance the reliability of the correlation coefficient. The *p*-value, which is less than 0.001, is well below the significance level of 0.05, underscoring the statistical significance of the findings. The R-squared value of 0.4145 indicates that approximately 41.45% of the variance in the anatomy test scores can be accounted for by the IQ scores. This is a substantial proportion, suggesting that IQ is a strong predictor of academic performance in this context. Additionally, the positive correlation coefficient of (r = 0.64) implies that as IQ scores increase, the anatomy test scores tend to increase as well, reinforcing the idea that higher intelligence is associated with better academic outcomes in anatomy.

**Figure 13:**
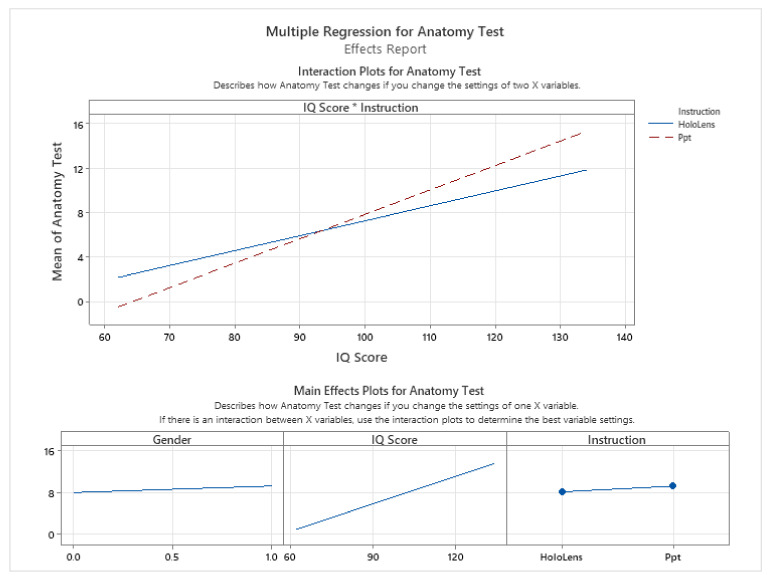
Influence of type of teaching material, gender, and IQ score on anatomy test results.

Next, we performed a multiple regression analysis to determine if more factors have an impact on the academic performance of the subject. Besides IQ score, we considered the instruction method and the gender of the participants. Figure 13 shows that out of these three variables, IQ score has the highest impact. 

Figure 13 presents an analysis that underscores the limited influence of gender on learning outcomes in various teaching settings. The data show a slight advantage for male participants over female participants; however, with a correlation coefficient of (r = 0.36) and an R-squared value of 13.22%, it is clear that gender does not play a significant role in the academic success of students, as measured by anatomy test scores, irrespective of the instructional method employed. The most influential variable affecting anatomy test results is the IQ score. This study further reveals that participants with an IQ score below 100 derive greater benefit from instructional methods utilizing mixed reality technology, such as the Microsoft HoloLens 2. On the other hand, participants with an IQ score above 100 appear to respond better to traditional educational materials presented through PowerPoint presentations. This suggests that the effectiveness of instructional methods may vary depending on the cognitive abilities of the learners.

**Figure 14:**
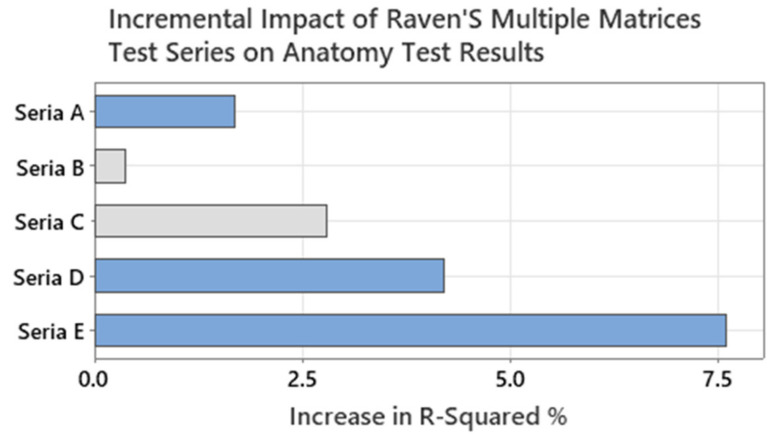
Regression for Raven’s Standard Progressive Matrix Test vs. anatomy test score.

A multiple linear regression analysis to examine the relationship between the scores of each series of the Raven’s Standard Progressive Matrix Test and the anatomy test results was also performed (see Figure 14). Raven’s test is a widely used measure of general intelligence that consists of five series of 12 questions, each with increasing difficulty and complexity. The series are labelled A, B, C, D, and E, and they assess different cognitive abilities such as observation, classification, analogy, reasoning, and synthesis. The E series questions are particularly challenging and require the ability to abstract and dynamically synthesize information from complex patterns and sequences. According to [71], the E series reflects the highest level of cognitive functioning and the most advanced stage of mental development. Therefore, we observed that the E series scores have the strongest correlation with the anatomy test results, as shown in Figure 14.

**Figure 15:**
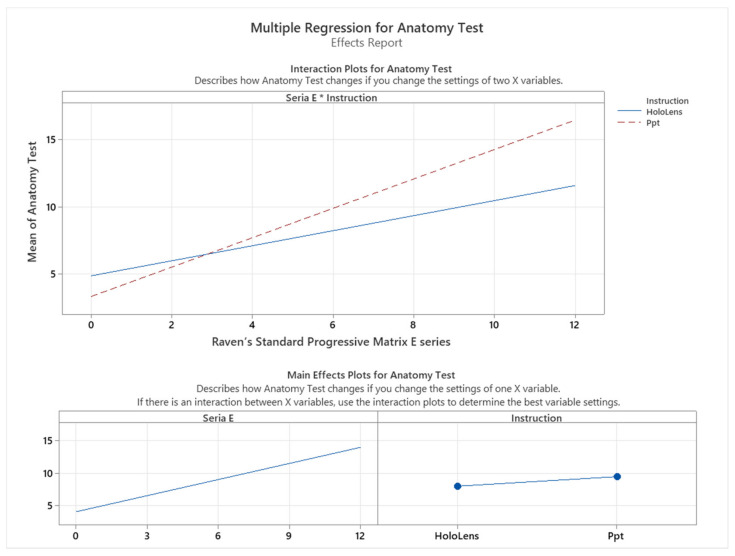
Multiple regression for Raven’s Standard Progressive Matrix E series vs. anatomy test score.

To further analyze the effect of the instruction method on the students’ performance, we conducted a multiple regression test for the series with the highest impact on the model, namely series E, as shown in Figure 15. The results of the multiple regression test indicated that there was a significant interaction between the instruction method and the number of correct answers to series E (*p* < 0.05). Specifically, we observed that the students who scored less than 3 out of 12 in series E performed better when instructed with HoloLens, while the students who scored more than 3 out of 12 in series E performed much better when instructed with traditional methods, namely PowerPoint as depicted in Figure 15. 

### 5.6. Does the High School Profile or Age of the Students Influence the Learning Outcomes?

The participants had graduated from five different high school profiles: Mathematics, Science, Individuals and Societies, Arts and Language, and Literature (see Figure 6). Statistical analyses were performed, and no correlations were observed. Therefore, the five profiles were further grouped into two categories: Mathematics and Sciences were grouped into a category titled “Sciences”, while the other three profiles were grouped into a category titled “Humanities”. 

We conducted a linear regression analysis to investigate the correlation between high school profiles and students’ learning outcomes. The regression model yielded the equation y = 7.741 + 1.6541x, with y representing the learning outcome score and x denoting the high school profile (1 = Sciences, 0 = Humanities). The coefficient of determination (R-squared) was calculated as 0.0359, indicating that merely 3.59% of the variance in learning outcomes can be accounted for by variations in high school profiles. The *p*-value associated with the slope was 0.062, suggesting a 6.2% likelihood of observing a slope as extreme as 0.01 or more extreme solely due to random chance. Consequently, we infer that the association between high school profiles and learning outcomes lacks statistical significance at the 0.05 level. This suggests that there is insufficient evidence to support the assertion that high school profiles influence students’ learning outcomes significantly or that such influence is minimal in comparison to other variables.

One of the factors that might affect the learning outcomes of the students is their age. To examine the relationship between the age of the students and their performance on the anatomy test, two types of regression analyses were conducted. The first one was a simple regression, where the age was the only predictor variable, and the post-test score was the outcome variable. The second one was a multiple regression, where the age, the instruction method and the IQ score were all included as predictor variables and the post-test score was again the outcome variable. By comparing these two analyses, it is possible to evaluate how much of the variance in the post-test score can be explained by the age alone and how much can be explained by the combination of the age and other factors (namely IQ score and instruction method).

### 5.7. Factors’ Influence over the Effectiveness of Learning

**Figure 16:**
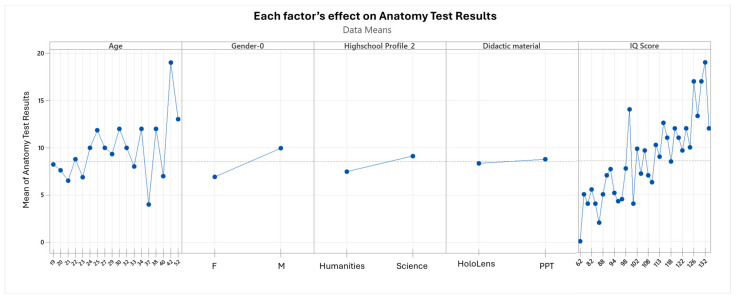
Each factor’s effect on the anatomy test results.

The analysis of factors influencing anatomy test performance, presented in Figure 16, highlights several key findings. Firstly, the correlation coefficient between test scores and student age is 0.28, indicating a very weak positive linear relationship. While there is a slight increase in test scores with age, it is not consistent or predictable. The *p*-value of 0.006 indicates a statistically significant difference between the test scores of younger and older students, but age itself is not a major explanatory factor.

Gender differences in academic performance are minimal, with male students slightly outperforming females, but the impact is not substantial.

High school profiles account for only 3.59% of the variability in academic outcomes, suggesting that high school background is not a significant determinant of anatomy test performance.

The most influential factor is IQ score, which shows a strong correlation with test results. Students with IQ scores below 100 benefit more from mixed reality technology, such as the Microsoft HoloLens 2, whereas those with higher IQ scores respond better to traditional PowerPoint presentations.

Furthermore, the E series scores from Raven’s Standard Progressive Matrix Test, which assess advanced cognitive abilities such as abstraction and synthesis, have the strongest correlation with anatomy test outcomes. This underscores the importance of cognitive functioning in academic performance, particularly in complex and dynamic learning environments.

## 6. Discussion

This study sought to investigate the effectiveness of MR technology compared to traditional pedagogical methods, such as PowerPoint presentations, in enhancing educational outcomes. Additionally, it examined the interplay between students’ Intelligence Quotient and the choice of educational medium. Our findings highlight the complex relationship between technological innovation, individual cognitive abilities, and learning outcomes.

*Effectiveness of MR vs. Traditional Methods:*

The results of our study revealed nuanced insights into the differential impacts of MR and traditional methods on learning outcomes [29,60]. Contrary to initial hypotheses, we did not find a clear superiority of MR over PowerPoint in improving educational performance. Instead, our findings suggest that the effectiveness of instructional methods may vary depending on individual characteristics, particularly IQ levels.

While MR offers immersive and interactive learning experiences, the novelty effect associated with this technology may influence participants’ engagement and attention [46]. 

*Role of IQ in Educational Efficacy:*

One of the contributions of our study is the exploration of IQ as a determinant of educational outcomes. Consistent with previous research [29,61,63], our findings suggest that students’ cognitive abilities significantly influence their response to different instructional methods. Participants with higher IQ scores demonstrated better performance when exposed to traditional PowerPoint presentations, while those with lower IQ scores benefitted more from MR-based learning environments.

The differential impact of IQ on learning outcomes highlights the need for personalized educational approaches [70] that cater to individual cognitive profiles. Educators and instructional designers should consider students’ cognitive strengths and weaknesses when selecting pedagogical strategies, leveraging technology to accommodate diverse learning preferences and abilities.

*Implications for Education:*

Our findings suggest that the use of innovative technologies, such as mixed reality (MR), is not sufficient to replace traditional methods like PowerPoint (PPT) presentations if the information provided remains the same. In our study, the MR device, HoloLens 2, displayed a static 3D model that users could only visually explore without any interactive capabilities. This limitation likely contributed to the lack of significant improvement in information retention compared to traditional PPT presentations. 

For MR technologies to fully realize their potential in enhancing learning outcomes, users need to be able to interact with the superimposed 3D models in meaningful ways—such as hiding, showing, exploding, manipulating, and extracting different parts of the model. Without these interactive elements, as demonstrated by our experiment, users may be captivated by the novelty of the technology but their retention of the information remains unchanged.

Moreover, our study underscores the necessity of adapting instructional methodologies based on users’ cognitive profiles. The results indicate that when the information provided is identical, the type of technology used (MR or PPT) does not significantly affect learning outcomes, as evidenced by the anatomy test scores. This highlights the importance of personalizing educational experiences to better suit individual cognitive abilities.

Future research should focus on developing and testing training scenarios that are tailored to each individual’s cognitive profile. Additionally, adaptive training scenarios, which adjust based on the user’s immediate feedback during continuous evaluation, are recommended. Such adaptive learning environments could potentially enhance engagement and retention by responding dynamically to the learner’s needs and progress.

By exploring the full interactive potential of MR technologies and customizing instructional approaches to fit cognitive profiles, we can better leverage these tools to improve educational outcomes. Further studies in these areas are essential to understand how best to integrate MR into diverse learning environments effectively.

This study also contributed to the existing literature on immersive technology in education, which has shown that AR and MR can create immersive and interactive learning environments that foster the motivation, collaboration, and creativity of learners [6,11,74]. AR and MR can also augment the physical world with digital content, such as 3D models or holograms, that can enhance the learning experience and facilitate comprehension of complex concepts [75]. 

## 7. Conclusions

This study aimed to investigate whether immersive technology, specifically MR, could improve the learning outcomes of students compared to traditional methods like PPT presentations. The literature review provides an overview of the current state of research on immersive technologies in education, indicating that while they can create engaging and interactive learning environments that enhance motivation, attention, and retention, there is a lack of empirical evidence on their effectiveness. 

Our study’s results demonstrate that students with higher IQ scores achieved better learning outcomes than those with lower IQ scores, regardless of the teaching method. This finding suggests that IQ is a more important predictor of learning outcomes than the teaching method itself. 

Furthermore, our research suggests that while innovative technologies like MR have potential, they cannot replace traditional methods like PPT if the content remains static and non-interactive. The use of HoloLens 2 with a static 3D model did not significantly improve information retention compared to PPT presentations, indicating the need for interactive capabilities to enhance learning outcomes. Additionally, our study emphasizes the importance of adjusting instructional strategies based on individual cognitive profiles, showing that the technology type (MR or PPT) does not significantly impact learning outcomes when the information is the same.

In conclusion, our study contributes to the growing body of research on immersive technologies and educational efficacy by highlighting the complex interplay between MR, IQ, and learning outcomes. We provide valuable insights for educators and technology developers seeking to optimize educational practices in the digital age. Future investigations should focus on designing personalized training scenarios for different cognitive profiles and creating adaptive learning environments that adjust to learners’ needs. By maximizing the interactive features of MR technologies and customizing instructional methods to match cognitive profiles, we can effectively enhance educational results. Further research is essential to understand the integration of MR into diverse learning settings and its implications for student learning and engagement.

### Study Limitations

Some of the limits of our approach are related to the fact that due to the anonymization of participants’ data, they can no longer be contacted to test how much information they retain several months after it has been provided to them. Although participants who used MR said they would enjoy learning through this technology, the motivation for learning could not be verified, requiring a long-term study. The sample should be extended to students from several fields (excluding medicine) and from different geographical locations. This study did not consider the type of learning that each individual prefers. Participants solved the tests after exams for organizational reasons. This can contribute to poor performance from respondents. The IQ test is lengthy (approx. 35 min), with many participants stating that it is “boring”. The sample consisted of 98 participants, which may not be enough to capture the diversity and complexity of the population under investigation. Moreover, the age range of the participants was wide, from 19 to 52 years old, which may introduce confounding variables and reduce the generalizability of the findings. A larger and more homogeneous sample, with a narrower age distribution, would have increased the statistical power and validity of the study.

Another limitation of this study is the potential influence of the novelty effect associated with MR technology on the cognitive capacity of participants. Given that none of the participants had prior experience with MR technology before this study, many were intrigued by the novel graphics and immersive experience provided by the MR device. As a result, some participants may have been more focused on exploring the technological features and visualizations rather than engaging deeply with the instructional material. This fascination with the MR environment could have led to a reduced attentional allocation towards the instructional content, potentially impacting their cognitive processing and learning outcomes. 

Moreover, while our study provides valuable insights into the effectiveness of MR and PPT in education, we acknowledge the limitations of our experimental design. To strengthen our findings, we propose incorporating a crossover study design in future research. Future research should also aim to recruit more participants and control for age-related factors.

## Figures and Tables

**Table 1:** Differences between VR, AR, and MR.

	Virtual Reality	Augmented Reality	Mixed Reality
Working environment	Artificial, totally immersive	Real, with digital objects superimposed	Artificial, but with the possibility of seeing the real environment
Required technology	VR glasses + proximity sensors	Smartphone or tablet	MR glasses
Interaction Type	VR controllers that enable interaction with the virtual environment	Touch screen gestures on your smartphone or tablet	Hand gestures, voice commands, focus

**Table 2:** User experience with immersive technologies.

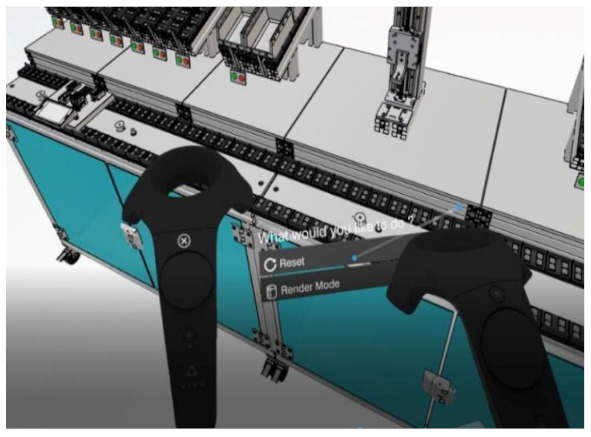	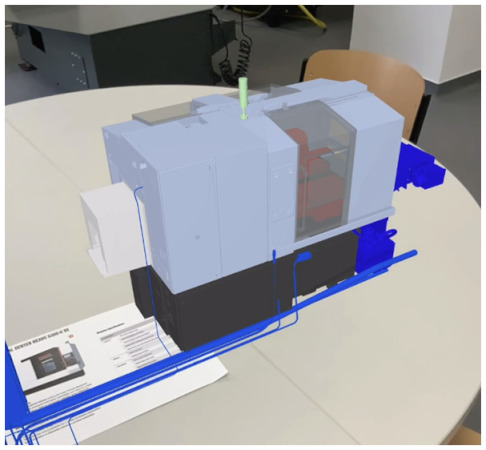	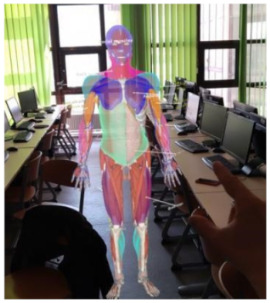
VR (original content)	AR [16]	MR (HoloAnatomy app Case Western Reserve University, n.d.) [17]

**Table 3:** Age of participants grouped by gender and instruction method.

Gender	Instruction Method	N	Mean	SE Mean	StDev	Minimum	Q1	Median	Q3	Maximum
Female	HoloLens	19	21.74	1.00	4.37	19.00	19.00	20.00	22.00	37.00
	Ppt	26	22.269	0.960	4.895	19.000	19.750	20.000	22.750	38.000
Male	HoloLens	32	23.406	0.865	4.891	19.000	21.000	22.000	23.000	40.000
	Ppt	21	25.48	2.03	9.28	19.00	19.00	20.00	31.00	52.00

**Table 4:** Item analysis for the 20 anatomy test questions.

Omitted Variable	Cronbach’s Alpha
1. The muscles of the upper limbs are:	0.7877
2. The muscles of the trunk include:	0.8055
3. The abdominal muscles are found:	0.7868
4. What muscles are shown in this picture?	0.7900
5. Identify the deltoid muscle in the following pictures:	0.7902
6. What muscles connect the trunk to the upper limb?	0.7935
7. Which of the following muscles are involved in walking?	0.7909
8. Which muscle is located closest to the gluteal muscles?	0.7801
9. Adjacent to the moss in the picture are:	0.7767
10. Which of the following muscles is inferior to the dorsal muscle?	0.7811
11. What muscle is activated when moving the upper limb?	0.7909
12. Which muscle is located inferior to the gluteal muscles?	0.7836
13. Which of the following statements are true?	0.7827
14. Which of the following claims are false?	0.7922
15. What is the correct order of muscles from head to toe?	0.7799
16. Which of the following muscles is not visible from a front view of the body?	0.7808
17. Which of the following muscles is visible from a back view of the body?	0.7846
18. What muscles are activated when we laugh?	0.8015
19. Identify the muscles of the upper limb.	0.7872
20. What is the correct order of muscles from the floor to the top of the head?	0.7835

## Data Availability

All data that were used for this research are available upon request.
